# Intake of Vegetables, Fruits and Berries and Compliance to “Five-a-Day” in a General Norwegian Population—The Tromsø Study 2015–2016

**DOI:** 10.3390/nu13072456

**Published:** 2021-07-18

**Authors:** Linn Nilsen, Laila A. Hopstock, Sameline Grimsgaard, Monica Hauger Carlsen, Marie W. Lundblad

**Affiliations:** 1 Department of Community Medicine, UiT The Arctic University of Norway, 9037 Tromsø, Norway; laila.hopstock@uit.no (L.A.H.); sameline.grimsgaard@uit.no (S.G.); marie.w.lundblad@uit.no (M.W.L.); 2 Department of Nutrition, Institute of Basic Medical Sciences, University of Oslo, 0372 Oslo, Norway; m.h.carlsen@medisin.uio.no

**Keywords:** population-based studies, food frequency questionnaire, food-based dietary guidelines, adults, recommendation adherence

## Abstract

Vegetables, fruits and berries are associated with reduced disease risk, and recommended intake is “five-a-day”. We studied the intake of vegetables, fruits and berries and compliance with “five-a-day” in 11,425 adults (40–96 years) who completed a food frequency questionnaire in the seventh wave of the Tromsø Study (2015–2016). Intake and proportion above/below recommended intake (250 g/day of vegetables and 250 g/day of fruits/berries, combined and separately) were analyzed. Logistic regression was used to examine compliance with recommendations in 10-year age-groups, and level of education, body mass index (BMI) and physical activity, in strata of sex and adjusted for total energy. Median intake of vegetables was 228 and 168 g/day, and fruits/berries 292 and 268 g/day, in women and men, respectively. In total, 31% of women and 17% of men met the five-a-day recommendation, and 44% and 60% of women, and 25% and 54% of men, met the recommendation for vegetables and fruits/berries, respectively. Odds of compliance with recommendation for both vegetables and fruits/berries were positively associated with age, education and physical activity (*p* trend < 0.001). The intake of vegetables, fruits and berries was suboptimal, in particular for vegetables. More women than men met the recommendation, and intake varied by education, physical activity level, age and BMI.

## 1. Introduction

Non-communicable diseases (NCDs), such as cardiovascular diseases, diabetes and cancer, are the leading cause of death globally and contributed to 73% of deaths in 2017. This number has increased by 23% from 2007 to 2017 and raises an urgent need to address the underlying causes [1]. Unhealthy diet is one out of three main risk factors for obesity and NCDs [2], and a low intake of vegetables and fruits, a high intake of added sugar, a high intake of saturated fat and a high intake of sodium are among the leading dietary risk factors for deaths and disability-adjusted-life years [3]. There is strong evidence that intake of vegetables, fruits and berries reduces the risk of, among others, obesity, coronary heart diseases and cancer in the digestive system [4].

In order to fight the epidemic of obesity and NCDs, an increased intake of vegetables, fruits and berries is advised. The World Health Organization (WHO) [5] recommends an intake of at least five servings—or 400 grams (g)—of fruits and vegetables a day. Food-based dietary guidelines are found in 90 countries worldwide, of which 84 of them have a key-message about fruit and vegetable intake [6], of which 30 are in accordance with the WHO recommendation [6]. The Norwegian food-based dietary guidelines recommend an intake of at least five servings a day (each equal 100 g), of which half (250 g) should be vegetables [7]. The recommendation includes fresh, hermetic, frozen and heat-treated vegetables, fruits and berries, and it is encouraged to vary between different types. Potatoes, legumes, grains, spices and herbs are not included in the Norwegian “five-a-day” recommendation.

Dietary surveys conducted among adults in the Nordic countries between 2010 and 2013 all found a suboptimal intake of vegetables, fruits and berries in the adult population [8,9,10,11]. The Norwegian national dietary survey NORKOST 3 (2010–2011) was conducted in 1787 adults 18–70 years, and found the mean intake of vegetables and fruits/berries to be 155 and 178 g/day, respectively [8]. Norwegian food-supply statistics found that the consumption of vegetables increased between 2010 and 2019, and the consumption of fruits and berries increased until 2015, and then had a slight decrease [12]. There is a need for more recent information on reported intake rather than food-supply statistics, including a large sample of adult and elderly participants [8]. This study presents data from one of the most recent surveys mapping diet in a general Norwegian population and may serve as an important foundation for comparison with both past and future studies presenting the intake of vegetables, fruits and berries.

The objective of this study was to present the intake of vegetables, fruits and berries; to investigate the association between sex, age, education, BMI and physical activity and intake; and, finally, to present the compliance with the Norwegian “five-a-day” recommendation in a general Norwegian population of adults and elderly.

## 2. Materials and Methods

### 2.1. Study Population

The Tromsø Study is a population-based ongoing cohort study consisting of seven completed surveys (Tromsø 1–Tromsø 7 1974–2016) [13] conducted in Tromsø municipality, consisting of both urban and rural populations. In Tromsø 7 (2015–2016), invitations were sent to all inhabitants aged 40 years or older in Tromsø municipality (*n* = 32,591). A total of 21,083 women and men (40–99 years) participated (65%) between March 2015 and October 2016. The data collection included biological sampling, clinical examinations including anthropometric measures and questionnaires, including a separate food frequency questionnaire (FFQ).

**Figure 1:**
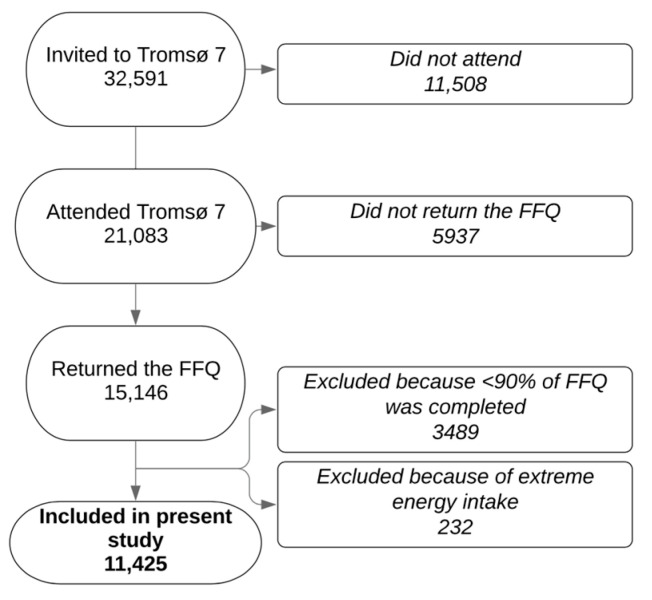
Flowchart of study sample. FFQ: food frequency questionnaire.

A total of 15,146 women and men completed the FFQ (72% of all Tromsø 7 participants). Participants with low completion rate (<90%) of the FFQ (*n* = 3489), and participants with unrealistic energy intakes (the 1% highest and lowest energy intake (above 21,267 or below 3948 kJ/da y, *n* = 232) were excluded (Figure 1), in accordance with Lundblad et al. [14]. Thus, the final sample in the present study included 11,425 persons aged 40–96 years (54% of all Tromsø 7 participants and 75% of those who completed the FFQ) (Table 1).

### 2.2. Sociodemographic and Anthropometric Information

Information on educational level (primary, secondary, tertiary short and tertiary long) and leisure-time physical-activity level (sedentary, light and moderate-to-vigorous) were included from a questionnaire. BMI (weight in kilograms (kg) divided by height in meters (m) squared) was calculated based on body weight and height measured by trained personnel and divided into three groups: normal (<25.0 kg/m^2^), overweight (25.0–29.9 kg/m^2^) and obese (≥30.0 kg/m^2^). Participants with underweight were merged with the normal-group because of few participants (*n* = 52).

### 2.3. Food Intake Measurements

An extensive previously validated [15] FFQ was used to collect information about diet during the past year. The FFQ consisted of 13 pages with questions about 261 different food items, dietary supplements, drinks (including alcoholic beverages) and meals (available in full version on the Tromsø Study website [16]). Intake of a variety of vegetables, fruits and berries was mapped by using questions on frequency and amount of intake: carrot, cabbage, turnip, cauliflower, broccoli, Brussel sprouts, onion (raw and fried), salad, paprika, avocado, tomato, corn, mix of frozen vegetables, mixed salad apple, pear, banana, orange, clementine, grapefruit, peach, nectarine, kiwi, grapes, melon, strawberries (fresh/frozen), raspberries, blueberries, cloudberries, raisins, dried fruit (e.g., apricot or figs), and fruit-and-nut mix. In addition, questions on numbers of daily servings of vegetables, fruits and berries were included. The food-and-nutrient calculation system Kostberegningssystemet (KBS), database AE14 (based on the Norwegian food composition tables 2014 and 2015), in software version 7.3, was used to calculate the intake of food, macro- and micronutrients at the University of Oslo. FFQ data collection and processing for Tromsø 7 have been described in detail elsewhere [14].

### 2.4. Data Analyses

Intake of vegetables, fruits and berries was investigated by calculating median (25th–75th percentile) intake and presenting the proportion of participants compliant with the “five-a-day” recommendation (Table 2 and Table 3). In addition, the participants were considered compliant or not with the recommendations for daily intakes of fruits/berries (at least 250 g) and vegetables (at least 250 g), respectively. We present median (25th–75th percentile) values rather than mean (standard deviation) values because the data were skewed to the right and thus were not normally distributed. We used multivariable linear regression to present the association between intake of vegetables, fruits and berries (g/day) and sociodemographic factors (Table 4). Logistic regression analyses were used to investigate the odds (odds ratio, OR) of compliance with recommendations (Table 5). All analyses were performed in strata of sex and in groups of 10-year age groups (40–49 years, 50–59 years, 60–69 years, 70–79 years or 80+years), education (primary, secondary, or low or high tertiary), BMI (normal < 25.0, overweight 25.0–29.9 or obese ≥ 30.0 kg/m^2^) and self-reported leisure-time physical activity (sedentary, light or moderate-to-vigorous), with the lowest group as reference group and mutually adjusted for 10-year age groups, education, BMI and self-reported leisure-time physical activity, respectively. The linear and logistic regression analyses were adjusted for total energy intake (kJ/day), and linear trends were investigated by including age, education, BMI and physical activity, respectively, as continuous variables in an identical analysis (Table 4 and Table 5). IBM SPSS v26 (IBM Corp. Released 2019. IBM SPSS for Macintosh, Version 26.0.0.1. Armonk, NY: IBM Corp) was used for all data analysis, and results were considered significant at a *p*-value of 0.001.

### 2.5. Ethical Considerations

The Tromsø Study was performed in accordance with the 1964 Helsinki declaration and its later amendments. Tromsø 7 was approved by the Regional Committee for Medical Research Ethics (REF North ref. 2014/940) and the Norwegian Data Protection Authority. All participants gave informed written consent.

## 3. Results

### 3.1. Study Population

A total of 11,425 persons were included in the analysis (53.4% women) (Table 1). About 50% of the participants had tertiary education. A total of 59.3% of women and 73.9% of men were overweight or obese, and about 13% reported doing sedentary activities during leisure time (Table 1). 

### 3.2. Five-a-Day

In total, 30.8% of women and 17.0% of men were compliant with the five-a-day recommendation (Table 2 and Table 3). The odds of being compliant with the five-a-day recommendation were positively associated with age, education, BMI and physical-activity level in both women and men (*p* linear trend < 0.001) (Table 5).

### 3.3. Intake of Vegetables

Median intake of vegetables was 228 and 168 g/day in women and men, respectively (Table 2 and Table 3). In total, 44.0% of women and 25.2% men were compliant with the recommendation (Table 2 and Table 3). Age, education, BMI and physical-activity level were positively associated with reported intake of vegetables in both women and men (*p* linear trend < 0.001) (Table 4). The odds of being compliant with the recommendation for vegetables increased with age, education, BMI and physical-activity level in both women and men (*p* linear trend < 0.001) (Table 5).

### 3.4. Intake of Fruits and Berries

Median intake of fruits/berries was 292 and 268 g/day in women and men, respectively, and 59.6% and 53.8% were compliant with the recommendation (Table 2 and Table 3). Age and education were positively associated with the intake of fruits/berries in both women and men (*p* linear trend < 0.001) (Table 4). The odds of being compliant with the recommendation for fruits/berries were positively associated with age, education and physical-activity level in both women and men (*p* linear trend < 0.001) (Table 5) 

## 4. Discussion

We found suboptimal intakes of vegetables, fruits and berries in the present study. Only 30.8% of women and 17.0% of men met the five-a-day recommendation; 44.0% of women and 25.2% of men met the recommended intake of 250 g vegetables per day; and 59.6% of women and 53.8% of men met the recommended intake of 250 g fruits/berries per day.

Overall, the reported intake of both vegetables, fruits and berries found in this study, conducted in 2015–2016, was higher than that found in women and men in national dietary surveys, conducted in 2010–2013, among adults in the Nordic countries Norway [8], Denmark [9] and Finland [11], and higher for women, but similar for men, in Sweden [10]. The higher reported intake found in this study, especially compared to the findings from the Norwegian survey NORKOST 3 conducted in 2010–2011 [8], can indicate that the intake in the population has increased during the period 2010–2011 to 2015–2016. This is supported by the annual report on the development in the Norwegian Diet from 2017, based on food supply statistics [17]. In the most recent report from 2019, however, the consume of vegetables, fruits and berries had decreased slightly from 2017 to 2018 [12].

A low proportion (30.8% of women and 17.0% of men) met the five-a-day recommendation. A study among persons aged 15 years and older in all member states of the European Union (EU) (The European Health Interview Survey (EHIS) 2013–2015) found that, on average, 14.1% reported a daily consumption of at least five fruits and vegetables [18]. This proportion did, however, vary across the EU, from around 25% in Denmark, the Netherlands, the United Kingdom and Ireland to less than 10% in Romania, Bulgaria, Croatia and Turkey [18].

In general, women reported higher intakes and were more compliant with the recommendation for both vegetables and fruits/berries, as compared to men. A similar higher reported intake among women than men were found in both the national dietary surveys from Nordic countries [8,9,10,11], in other population-based studies in Norway [16,17] and in the large survey from the EU (EHIS) [18].

We found age to be positively associated with reported fruit and vegetable intake in women and men. A similar gradient (although weak) was found in other Norwegian population-based studies [19,20]. Education was positively associated with the reported intake of vegetables, fruits and berries in both women and men. This corresponds to findings from previous Nordic national dietary surveys, Nordic population-based studies and the large survey from the EU [8,10,18,19,20,21]. Obesity was positively associated with reported vegetable intake in both women and men. This is supported by similar findings from the SAMINOR2 study [22], but it contrasts with the findings from the Swedish national dietary survey [10]. Physical activity was positively associated with the reported intake of vegetables, fruits and berries in both women and men, corresponding to similar findings in the Swedish national dietary survey [10] and in population-based surveys from Norway and Sweden [19,21].

Education is frequently used as an appropriate indicator for socioeconomic status [23,24], and the association between education and intake of fruit and vegetables found in this study may serve as a measure of social inequality in health. An educational gradient in favor of those with higher education was also found for intake of fiber, proteins and added sugar in a previous study of the same population [25].

These results are important for future studies investigating total intake and trends in fruit and vegetable intake in general populations and for researchers interested in comparing results across different populations. Future studies are warranted for observing potential changes in fruit and vegetable intake, and further to explain why differences in subgroups occur. It would also be useful to explore those with a low intake of fruit and vegetables further to attain a deeper understanding of the mechanisms behind the low intake, and to investigate whether they have other dietary characteristics that are unfavorable for the health.

### Strengths and Limitations

This study had a large and population-based sample of adult and elderly women and men from both urban and rural living areas, representative of the general Norwegian population as for the distribution of sex, age, educational attainment, BMI and moderate-to vigorous activity level [26,27,28]. However, although the overall participation was high (65%), selection bias is possible. Previous studies from health surveys in Norway have indicated that participants in health surveys tend to have higher educational attainment and better health than non-responders [29,30,31]. However, Lundblad et al. [14], using the same sample as in the present study, concluded that Tromsø 7 attenders were similar to the non-attenders. Thus, the external validity in this study is probably high. 

Another strength is that the FFQ used for data collection is previously validated for several dietary factors in studies of Norwegian adults [15,32,33]. However, a general limitation of the use of a FFQ is the risk of misclassification due to inaccurate memory or for instance social desirability bias. Social desirability bias would imply that some participants might over-report their intake of healthy food items, such as vegetables, fruits and berries [34]. The inclusion/exclusion criteria of ≥90% completeness of the FFQ and the exclusion of highly unrealistic energy intakes ensured that some of the cases with high risk of uncertainty in the dietary assessment were removed.

## 5. Conclusions

In this Norwegian population-based sample of adults and elderly, the median intake of vegetables, fruits and berries was suboptimal. This applies especially to the intake of vegetables. More women (31%) than men (17%) met the five-a-day recommendation. Furthermore, 44% of women and 25% of men met the recommended intake of 250 g vegetables per day and approximately 57% met the recommended intake of 250 g/day of fruits/berries per day. Odds of compliance with recommendation for both vegetables and fruits/berries were positively associated with age, education and physical-activity level.

## Figures and Tables

**Table 1:** Study participants. The Tromsø Study 2015–2016.

	Women	Men
Age, years	56.9 (10.7)	58.1 (11.0)
Age group (%)		
40–49 years	30.0 (1833)	26.9 (1433)
50–59 years	29.7 (1813)	27.5 (1464)
60–69 years	27.0 (1646)	28.6 (1521)
70–79 years	11.1 (679)	14.4 (768)
80–96 years	2.2 (133)	2.5 (135)
Education level ^a^ (%)		
Primary	20.8 (1270)	19.9 (1057)
Secondary	25.2 (1539)	28.7 (1525)
Tertiary short	18.2 (1112)	22.7 (1209)
Tertiary long	34.8 (2122)	27.6 (1468)
Body mass index (%)		
Normal (<24.9 kg/m^2^)	40.4 (2466)	26.0 (1382)
Overweight (25.0–29.9 kg/m^2^)	37.5 (2288)	50.4 (2680)
Obese (≥30.0 kg/m^2^)	21.8 (1331)	23.5 (1248)
Physical activity level ^b^ (%)		
Sedentary	12.3 (751)	13.2 (704)
Light	63.2 (3857)	50.7 (2700)
Moderate-to-vigorous	21.7 (1327)	34.2 (1822)

Numbers are mean (standard deviations) or percentages (total number). ^a^ Primary (up to 10 years of schooling); secondary education (a minimum of 3 years); tertiary short (college/university less than 4 years); tertiary long (college/university 4 years or more). ^b^ Exercise and physical activity in leisure time over the last year: sedentary (reading, watching TV/screen or other sedentary activity); light (walking, cycling or other forms of exercise at least 4 h a week); moderate-to-vigorous (participation in recreational sports, heavy gardening, snow shoveling, etc., at least 4 h a week or participation in hard training or sports competitions, regularly, several times a week).

**Table 2:** Median intake of vegetables, fruits and berries in women, and proportion compliant with recommendations. The Tromsø Study 2015–2016.

	Vegetables			Fruits/berries			Five-a-Day
Characteristic	Median (g/day)	25th–75th Percentile	Intake of ≥250 g/day (%)	Median (g/day)	25th–75th Percentile	Intake of ≥250 g/day (%)	Intake of ≥250 g vegetables and ≥250 g fruits/berries a day (%)
Total	228	148–330	44.0	292	180–445	59.6	30.8
Age Group (years)							
40–49	223	146–327	41.8	273	162–428	55.0	27.6
50–59	240	159–341	47.4	295	184–452	40.2	33.1
60–69	232	150–332	45.7	303	196–443	37.8	32.1
70–79	207	131–306	38.0	312	196–487	36.2	29.6
80–96	193	122–285	35.3	334	205–493	65.4	30.1
Education level ^a^							
Primary	197	121–289	35.3	268	167–402	54.2	23.5
Secondary	226	145–327	43.3	286	168–453	57.5	29.5
Tertiary short	226	149–328	42.8	291	179–436	59.9	30.6
Tertiary long	251	165–352	50.3	312	201–467	64.1	36.1
Body mass index (kg/m^2^)							
Normal (<25.0)	231	152–333	44.9	301	182–462	60.6	32.4
Overweight (25.0–29.9)	223	148–323	42.4	291	189–443	60.0	29.5
Obese (≥30.0)	231	142–334	44.9	279	170–423	56.9	30.0
Physical activity level ^b^							
Sedentary	185	108–274	31.8	237	134–380	47.4	19.2
Light	229	150–326	43.9	290	181–441	59.5	30.1
Moderate-to-vigorous	259	170–372	52.1	325	216–490	66.7	39.5

^a^ Primary (up to 10 years of schooling); secondary education (a minimum of 3 years); tertiary short (college/university less than 4 years); tertiary long (college/university 4 years or more). ^b^ Exercise and physical activity in leisure time over the last year: sedentary (reading, watching TV/screen or other sedentary activity); light (walking, cycling or other forms of exercise at least 4 h a week); moderate-to-vigorous (participation in recreational sports, heavy gardening, snow shoveling, etc., at least 4 h a week or participation in hard training or sports competitions, regularly, several times a week).

**Table 3:** Median intake of vegetables, fruits and berries in men, and proportion compliant with recommendations. The Tromsø Study 2015–2016.

	Vegetables			Fruits/berries			Five-a-Day
Characteristic	Median (g/day)	25th–75th Percentile	Intake of ≥250 g/day (%)	Median (g/day)	25th–75th Percentile	Intake of ≥250 g/day (%)	Intake of ≥250 g/day of vegetables and ≥250 g fruits/berries a day (%)
Total	168	103–251	25.2	268	155–426	53.8	17.0
Age Group (years)							
40–49	171	108–250	24.8	258	141–424	51.5	16.1
50–59	172	106–262	27.2	261	146–422	52.4	17.8
60–69	164	101–247	24.3	271	164–432	55.2	16.4
70–79	163	100–243	23.6	282	169–420	56.8	17.7
80–96	148	90–265	25.9	285	162–473	59.3	19.3
Education level ^a^							
Primary	136	80–216	18.5	237	127–387	47.1	12.1
Secondary	163	99–241	23.1	245	138–401	59.3	14.7
Tertiary short	172	110–259	26.6	274	161–430	54.8	18.0
Tertiary long	188	122–278	30.7	314	192–474	62.6	21.9
Body mass index (kg/m^2^)							
Normal (<25.0)	164	101–250	25.2	281	169–441	57.2	17.2
Overweight (25.0–29.9)	168	105–248	24.4	267	154–431	53.3	16.8
Obese (≥30.0)	174	102–260	26.8	253	142–404	50.9	17.1
Physical activity level ^b^							
Sedentary	137	81–208	16.8	216	104–363	44.3	8.8
Light	167	103–250	16.8	263	156–424	52.7	16.7
Moderate-to-vigorous	185	118–274	25.0	298	180–458	59.2	20.9

^a^ Primary (up to 10 years of schooling); secondary education (a minimum of 3 years); tertiary short (college/university less than 4 years); tertiary long (college/university 4 years or more). ^b^ Exercise and physical activity in leisure time over the last year: sedentary (reading, watching TV/screen or other sedentary activity); light (walking, cycling or other forms of exercise at least 4 h a week); moderate-to-vigorous (participation in recreational sports, heavy gardening, snow shoveling, etc., at least 4 h a week or participation in hard training or sports competitions, regularly, several times a week).

**Table 4:** Linear regression analysis of intake of vegetables and fruits/berries. The Tromsø Study 2015–2016.

	Vegetables		Fruits and Berries	
	Women	Men	Women	Men
Age group (years)				
40–49	Reference			
50–59	28 ** (18, 38)	11 * (2, 20)	47 ** (30, 62)	6 (−13, 26)
60–69	36 ** (26, 47)	16 ** (7, 25)	63 ** (47, 80)	50 ** (30, 70)
70–79	20 * (6, 34)	21 ** (11, 32)	103 ** (81, 126)	78 ** (53, 102)
80–96	15 (−11, 42)	31 * (9, 52)	132 ** (88, 175)	101 ** (53, 150)
*p* linear trend	<0.001	<0.001	<0.001	<0.001
Education level ^a^				
Primary	Reference			
Secondary	17 * (6, 28)	18 ** (8, 27)	28 * (10, 46)	7 (−18, 17)
Tertiary short	23 ** (11, 35)	27 ** (18, 37)	25 * (5, 45)	25 * (3, 47)
Tertiary long	41 ** (30, 52)	42 ** (32, 52)	39 ** (20, 57)	62 ** (40, 83)
*p* linear trend	<0.001	<0.001	<0.001	<0.001
Body Mass Index (kg/m^2^)				
Normal (<25.0)	Reference			
Overweight (25.0–29.9)	5 (−2, 15)	9 * (1, 17)	−4 (−26, 7)	−1 (−18, 17)
Obese (≥30.0)	20 ** (10, 29)	24 ** (15, 33)	−10 (−26, 7)	9 (−13, 30)
*p* linear trend	<0.001	<0.001	0.5	0.2
Physical activity level ^b^				
Sedentary	Reference			
Light	33 ** (22, 44)	28 ** (19, 38)	16 (−2, 34)	31 (9, 52)
Moderate-to-vigorous	58 ** (45, 71)	39 ** (29, 49)	49 ** (28, 70)	44 ** (21, 68)
*p* linear trend	<0.001	<0.001	0.02	0.03

Results are adjusted for total energy intake (kJ/day) and given as unstandardized B (95% confidence interval). A separate analysis was performed for age groups, education level, body mass index and physical-activity level, respectively, with adjustment for all other covariates. * Statistically significant (*p* < 0.05). ** Statistically significant (*p* < 0.001). ^a^ Highest level of educational attainment: primary = up to 10 years of schooling, secondary education = a minimum of 3 years, tertiary short = college/university less than 4 years and tertiary long = college/university 4 years or more. ^b^ Exercise and physical activity in leisure time over the last year: sedentary = reading, watching TV/screen or other sedentary activity; light = walking, cycling or other forms of exercise at least 4 h a week; moderate-to-vigorous = participation in recreational sports, heavy gardening, snow shoveling, etc., at least 4 h a week or participation in hard training or sports competitions, regularly, several times a week.

**Table 5:** Logistic regression analysis of odds of being compliant with recommendations. The Tromsø Study 2015–2016.

	Vegetables		Fruits and Berries		Five-a-Day	
Recommendation (NNR 2012)	Intake of ≥250 g/day				Intake of ≥250 g of vegetables and ≥250 g fruits/berries a day	
	Women	Men	Women	Men	Women	Men
Age group (years)						
40–49	Reference					
50–59	1.5 ** (1.3, 1.7)	1.3 * (1.1, 1.4)	1.5 * (1.3, 1.7)	1.2 (1.1, 1.5)	1.7 ** (1.4, 1.9)	1.3 * (1.1, 1.6)
60–69	1.7 ** (1.4, 1.9)	1.3 * (1.1, 1.6)	2.0 ** (1.7,2.3)	1.7 ** (1.5, 2.0)	2.0 ** (1.7, 2.3)	1.6 ** (1.3, 2.0)
70–79	1.3 * (1.1, 1.6)	1.5 ** (1.2, 1.9)	2.7 ** (2.2, 3.3)	2.3 ** (1.9, 2.8)	2.0 ** (1.6, 2.6)	2.2 ** (1.7, 2.8)
80–96	1.4 (0.9, 2.2)	2.1 * (1.4, 3.3)	3.1 ** (2.0, 4.8)	2.9 * (1.9, 4.3)	2.5 ** (1.6, 4.0)	2.9 ** (1.8, 4.9)
*p* linear trend	<0.001	<0.001	<0.001	<0.001	<0.001	<0.001
Education level ^a^						
Primary	Reference					
Secondary	1.3 * (1.1, 1.6)	1.4 * (1.1, 1.7)	1.2 (1.0, 1.4)	1.1 (1.0., 1.3)	1.3 * (1.1, 1.6)	1.4 * (1.1, 1.7)
Tertiary short	1.3 * (1.1, 1.6)	1.6 ** (1.3, 2.0)	1.4 ** (1.1, 1.7)	1.4 ** (1.2, 1.7)	1.5 ** (1.2, 1.8)	1.7 ** (1.3, 2.2)
Tertiary long	1.8 ** (1.5, 2.0)	2.0 ** (1.7, 2.5)	1.6 ** (1.4,1.9)	2.0 ** (1.7, 2.4)	1.9 ** (1.6, 2.3)	2.2 ** (1.7, 2.9)
*p* linear trend	<0.001	<0.001	<0.001	<0.001	<0.001	<0.001
Body mass index (kg/m^2^)						
Normal (<25.0)	Reference					
Overweight (25.0–29.9)	1.0 (0.9, 1.1)	1.1 (0.9, 1.3)	1.0 (0.9, 1.2)	1.0 (0.8, 1.0)	1.0 (0.9, 1.1)	1.2 (1.0, 1.4)
Obese (≥30.0)	1.2 * (1.1, 1.4)	1.4 ** (1.2, 1.7)	1.0 (0.9, 1.2)	1.0 (0.8, 1.1)	1.1 (1.0, 1.3)	1.4 * (1.1, 1.8)
*p* linear trend	<0.001	<0.001	0.7	0.7	0.06	0.009
Physical activity level ^b^						
Sedentary	Reference					
Light	1.6 ** (1.3, 1.9)	1.5 ** (1.2, 1.9)	1.5 ** (1.3, 1.8)	1.3 * (1.1, 1.5)	1.7** (1.4, 2.1)	2.0 ** (1.5, 2.6)
Moderate-to-vigorous	2.1 ** (1.7, 2.6)	1.7 ** (1.4, 2.2)	2.1 ** (1.7, 2.5)	1.6 ** (1.3, 1.9)	2.5 ** (2.0, 3.2)	2.4 ** (1.8, 3.2)
*p* linear trend	<0.001	<0.001	<0.001	<0.001	<0.001	<0.001

Results are adjusted for total energy intake and given as odds ratio (95% confidence interval). A separate analysis was performed for age groups, education level, body mass index and physical-activity level, respectively, with adjustment for all other covariates. * Statistically significant (*p* <0.05). ** Statistically significant (*p* <0.001). ^a^ Highest level of educational attainment: primary, up to 10 years of schooling; secondary education, a minimum of 3 years; tertiary short, college/university less than 4 years; tertiary long, college/university 4 years or more. ^b^ Exercise and physical activity in leisure time over the last year: sedentary = reading, watching TV/screen or other sedentary activity; light = walking, cycling or other forms of exercise at least 4 h a week; moderate-to-vigorous = participation in recreational sports, heavy gardening, snow shoveling, etc., at least 4 h a week or participation in hard training or sports competitions, regularly, several times a week.
